# Enhanced conspicuousness of prey in warmer water mitigates the constraint of turbidity for predators

**DOI:** 10.1093/beheco/araf079

**Published:** 2025-07-10

**Authors:** Costanza Zanghi, Jolyon Troscianko, Christos C Ioannou

**Affiliations:** School of Biological Sciences, Life Sciences Building, University of Bristol, Tyndall Avenue, Bristol, BS8 1TQ, United Kingdom; Faculty of Health and Life Sciences, Stella Turk Building, University of Exeter, Penryn Campus, Penryn, TR10 9FE, United Kingdom; School of Biological Sciences, Life Sciences Building, University of Bristol, Tyndall Avenue, Bristol, BS8 1TQ, United Kingdom

**Keywords:** encounter, multi-stressors, multiple stressors, optical flow, *Poecilia reticulata*, predator-prey interactions, temperature, vision

## Abstract

Changes in environmental conditions impact predator-prey interactions by altering behavior through sensory and non-sensory (eg metabolic or cognitive) pathways. Elevated water temperature and turbidity are known to alter activity levels and anti-predator responses in prey fish, and are increasing globally as a result of anthropogenic activities. Less is known about how temperature and turbidity impact predators’ ability to detect prey directly, or indirectly via changes to prey behavior. We quantified the detectability of Trinidadian guppies (*Poecilia reticulata*) free-swimming in a large arena from the perspective of a stationary visual predator (simulated as an underwater camera). We used a fully factorial experimental design testing the independent and combined effects of increased temperature and turbidity. We found that both stressors had a strong influence on the appearance of prey (objectively quantified as the mean magnitude of the optical flow in the videos). As expected, turbidity reduced the frequency of detection between the guppies and the simulated predator, ie the magnitude of optical flow exceeded the threshold for a “detection event” more often in clear water. Events were also shorter in duration in turbid water, reducing the time available for a predator to detect the prey. However, during an event, prey were more detectable in warmer water (ie the mean magnitude was greater). Although we found no evidence of interactive effects of turbidity and temperature on the response variables, their cumulative main effects suggest an antagonistic effect between the two stressors on the predator-prey dynamic overall.

## Teaser

In warmer, murky water, prey fish may encounter predators less often, but when they do, they are at a higher risk of detection. Warmer water enhances movement in prey fish, making them easier to spot during these encounters, despite the poor visual conditions.

## Introduction

An ambush predator must first encounter, detect, and recognize potential prey items before launching an attack (Lima and Dill 1990). Encounters are determined by the rate at which predator and/or prey are within the sensory range of the other. Studying encounter rates thus often involves exploring the factors that allow a predator to get within physical proximity to their prey, and conversely, the avoidance behaviors of prey that minimize encountering predators (Ioannou et al. 2008; Ferrero et al. 2011). Detection is the sensory process that happens within an encounter, resulting in the identification and localization of an object (the signal) that is not part of its surrounds (the noise) (Troscianko et al. 2009). Once detected, the object must be classified as something of interest, which can either result in successful recognition of the object, or misidentification (as occurs with mimicry or masquerade, Ruxton et al. 2018). Each of the steps in the predator-prey interaction sequence (Lima and Dill 1990) can be influenced by various factors including the hunting mode of predators (Sims et al. 2008; Wearmouth et al. 2014), the prey’s foraging and anti-predator strategies (Magurran 1990; Turesson et al. 2009; Carroll and Sherratt 2013), other predator and prey traits (Harris et al. 2010; Szopa-Comley et al. 2020; Franco et al. 2022) and abiotic environmental conditions (Abrahams et al. 2007; Domenici et al. 2019).

Environmental conditions can impact encounter rates by altering how predators and prey use and move through their environment. For example, turbidity can alter the search behavior of diving seabirds that use vision to pursue prey underwater (Darby et al. 2022). Similarly, bat species whose prey-detecting echolocation calls overlap in frequency with sources of anthropogenic acoustic noise reduce activity around such sources (Bunkley et al. 2015). When predator and prey become close enough that at least one can detect the other, ie an encounter occurs, environmental conditions can affect the probability of detection by masking sensory cues. For instance, visual predators such as the three-spined stickleback (*Gasterosteus aculeatus*) were less likely to respond to prey in environments with increased visual noise (ie caustics; Attwell et al. 2021). Similarly, environmental conditions can distract individuals, impairing their ability to assess their surroundings efficiently. This distraction can weaken anti-predator responses, as seen in hermit crabs (*Coenobita clypeatus*) when exposed to both acoustic and visual noise (Chan et al. 2010), or in dwarf mongooses (*Helogale parvula*), which were slower to respond to secondary predator cues under anthropogenic acoustic noise (Morris-Drake et al. 2016). Increased turbidity can impact predator-prey interactions by both masking visual cues, reducing detection distances (Nieman and Gray 2019), disrupting shoal dynamics (Borner et al. 2015), and diminishing prey’s risk awareness, thereby distracting them from threats (Gregory 1993). Additionally, environmental parameters that affect the physiology and behavior of prey can render them more or less conspicuous to their predators. Predators detect prey more easily when their activity levels are raised due to higher water temperatures (Krause and Godin 1995), because predators have often evolved to be highly sensitive to prey movement (Ioannou and Krause 2009), and movement is more readily detectable than other visual information (Troscianko et al. 2009). This explains why, for instance, after being detected by a predator, freezing is a common response observed in many taxa (Eilam 2005).

Little is known about how the interactions between multiple environmental stressors affect encounter and detection in predator-prey interactions. This is important because rapid environmental change is affecting predator-prey interactions at a global scale (Abrahams et al. 2007; Domenici et al. 2007). Environmental stressors, here defined as any parameter experienced by a system with increased frequency and intensity (Townsend et al. 2008), rarely impact biological processes in isolation. Instead, they co-occur, leading to cumulative impacts on a system (ie additive effects). Additionally, these stressors can interact in ways that cause “ecological surprises” (Paine et al. 1998). In such cases, the resulting impact is unpredictable based on the responses to each stressor in isolation, resulting in an overall response that is greater (synergism) or lesser (antagonism) than the sum of the individual responses (Folt et al. 1999).

In this study, we adopted an approach developed by Turesson and Brönmark (2007), who assessed encounter rates between prey fish and a side-view facing underwater video camera, which was used to quantify prey appearance from the perspective of an aquatic ambush predator. Based on their observations in natural lakes, Turesson and Brönmark (2007) found that eutrophication and water transparency had important effects on encounter rates. In more turbid conditions, the distance at which prey were visible was so impaired that the probability of encountering prey decreased, even when prey density was at its highest. While Turesson and Brönmark (2007) directly observed video recordings to extract behavioral metrics such as camera detection distance, the frequency of encounter, encounter duration and shoal size, here we used optical flow analysis to objectively estimate the apparent motion of prey fish from the video footage as a measure of the prey’s likelihood of being encountered and then detected by a predator. Optical flow analysis is a computer-vision tool widely used for detecting and quantifying motion (Sun et al. 2014). Its application in biology spans diverse fields, including the assessment of farmed animal welfare (Dawkins et al. 2009, 2012), the investigation of animals’ self-motion and spatial awareness (Alexander et al. 2022), and the analysis of footage from wild animal camera traps (Khorrami et al. 2012). Optical flow allowed us to quantify not only the frequency and duration that prey could be detected (as in Turesson and Brönmark (2007)), but also how conspicuous to visual predators prey are during these encounters. All of these factors can be considered to contribute to the detectability of prey, and be likely indicative of the frequency of attacks by predators and hence wider ecological impacts of predation (Lima 1998; Chase et al. 2002).

To investigate the effects of changing environmental conditions on prey detectability, we designed a fully factorial laboratory experiment. We used free-swimming Trinidadian guppies (*Poecilia reticulata*) under control and elevated water temperatures and turbidities, as perceived by a hypothetical stationary visual predator. Increased turbidity reduces light levels and visual contrast, limiting the detection abilities of predators (Beauchamp et al. 1999; Vogel and Beauchamp 1999; Hansen et al. 2013). In addition to the direct effect of turbidity on visibility underwater, these stressors can have contrasting effects on the activity and shoaling behavior of guppies, which, in turn, impact encounter rates. The shoaling behavior of guppies in warmer temperatures have been observed to vary, increasing or decreasing, depending on the level of perceived risk (Weetman et al. 1998, 1999). Likewise, other studies have reported varying effects of turbid water on guppies' activity levels, with some finding no effect (Zanghi et al. 2023), while others have observed heightened or lessened activity (Borner et al. 2015; Ehlman et al. 2015).

Building on this previous literature, we hypothesized that in warmer water, guppies would be more active and hence more frequently observed within the camera's field of view. This would, however, reduce the amount of time over which detection could occur for each event (limiting the probability of detection per event). Under turbid conditions, detection distances are reduced, but moving targets closer to the camera within its field of view are still detectable. Thus, while encounter frequency would be reduced, detection probability at closer distances would increase. In the interaction treatment, we hypothesized a synergistic effect, where guppies entering the camera's field of view would be more detectable compared to the additive effect of turbid and warmer water in isolation. However, if temperature and turbidity have opposing effects on activity, in the interaction treatment there may be an antagonistic effect, where the detectability is similar to the control treatment of clear and ambient temperature water.

## Methods

### Experimental set up

**Fig. 1.**
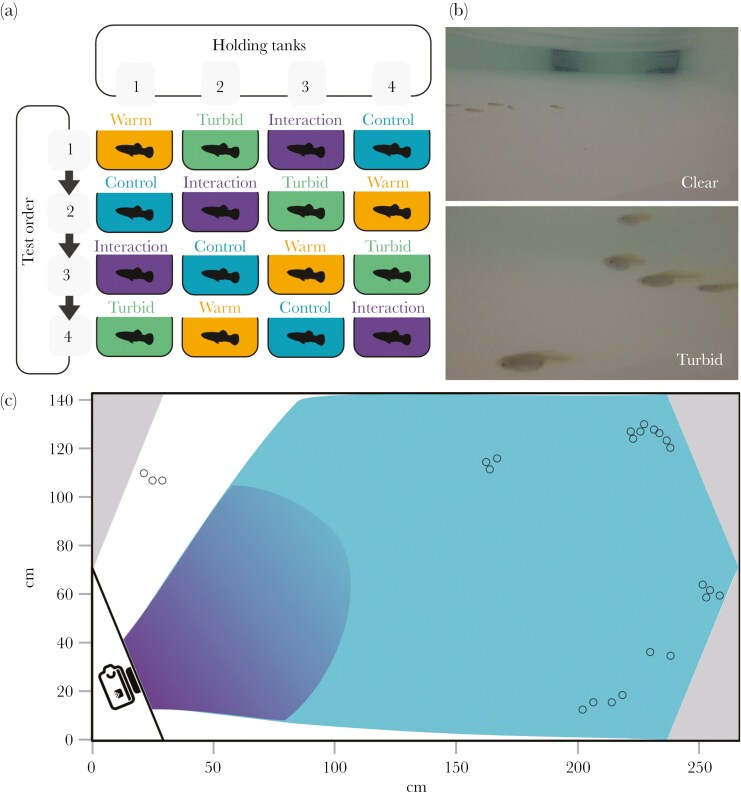
Protocol detailing the balanced crossover Latin square design for treatment order **(a)**: control (0NTU, 22 °C), turbid (5NTU, 22 °C), warm (0NTU, 29 °C), and interaction (5NTU, 29 °C). **(b)** Camera views in clear and turbid water. **(c)** Schematic of the experimental arena showing refuges housing the heaters (gray corners), example of fish positions in a single frame (open circles), and the transparent divider (black line) for the camera placement. The lighter blue area represents the field of view of the camera in clear water, the darker blue in turbid water. Adapted from Allibhai et al. 2023.

The data presented in this study was collected during a parallel study by Allibhai et al. (2023), where details on the provenance and housing of the study subjects are described. In brief, 720 adult, mixed-sex (5:2 female to male ratio) guppies (*Poecilia reticulata*) were haphazardly assigned to four 200 L holding tanks. Over 16 d between February and March 2021, all guppies were tested once under each of four experimental treatments: control (same as holding conditions: 0 Nephelometric Turbidity Units, NTU and 22 °C), increased turbidity (5 NTU, 22 °C), warmer temperature (0 NTU, 29 °C), and a combined turbid and warmer treatment (5 NTU, 29 °C). These conditions reflect the natural variation observed in the guppies’ native habitat in Trinidad (Zanghi et al. 2024). The sequence of treatments was arranged in a balanced crossover Latin square design to reduce order effects (Fig. 1a; N_trial_ = 91; five trials (two warm, two turbid, and one in the interaction treatment) were excluded as the camera malfunctioned during filming).

For each trial, approximately 30 fish (26 to 32 individuals per group; the small difference was due to human error) were haphazardly caught with hand nets from the holding tank and gently released in the middle of the experimental arena (Fig. 1c, 255 × 140 cm, with 10 cm water depth). Testing nearly all individuals from each holding tank each day, and maintaining large sample sizes per tank, minimized between-group variation in sex ratio and other sources of inter-individual differences. Fish were filmed from the side view for 15 min using an underwater camera (GoPro Hero5) placed behind a transparent divider in one corner of the tank. Trials were recorded in 2.7K resolution (2704 × 1520 pixels), in wide mode, and at a frame rate of 30 frames per second. Lighting was provided by fluorescent tubes above the center of the arena, diffused by white cloth over the test tank throughout the experiment. The light intensity was 270 lux, as measured with an in-water sensor (HOBO-MX2202) during a pilot trial.

Turbidity was achieved using dissolved kaolin clay powder (Fig. 1b) and it was measured using a turbidity meter (Thermo Scientific Orion AQUAfast AQ3010) before and after each trial with increased turbidity. Temperature was manipulated by using submersible heaters placed in the corner of the experimental arena, and it was measured using a thermometer before and after each trial. The pilot trial and the monitoring before and after each experimental trial showed that both temperature and turbidity levels remained within appropriate parameters throughout the experiment and across the large arena (±0.5 °C for temperature and ±1.5 NTU for turbidity). To maintain consistency, the water was thoroughly mixed by hand and aerated with oxygen stones between trials. Approximately every 2 d, the tank was drained, cleaned, and replenished with fresh aged water.

### Data extraction

**Fig. 2.**
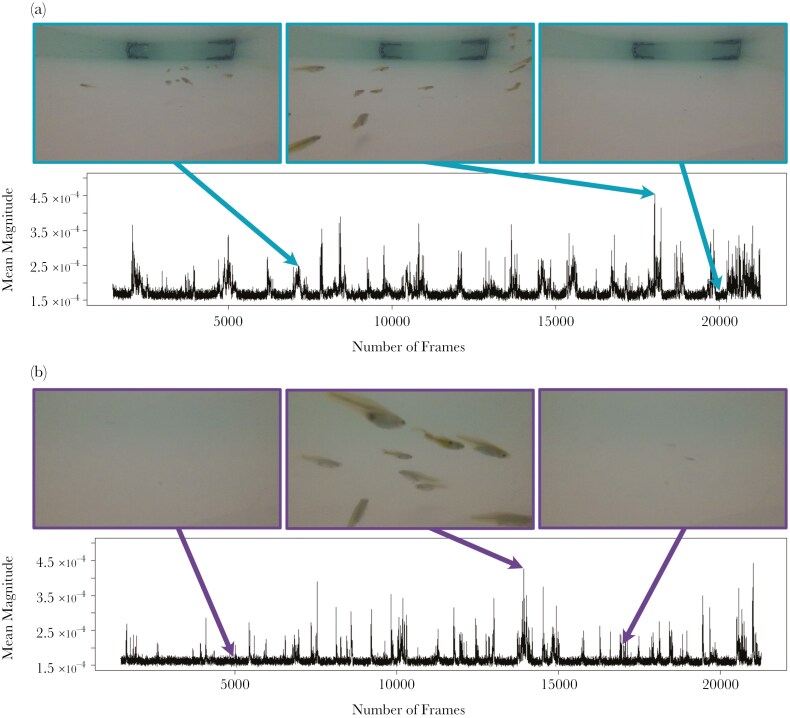
**Example from two trials, for the control (a) and interaction (b) treatment.** In the time series, the dark band at low mean magnitudes represents background noise, while spikes in mean magnitude represent frames in which fish motion was detected. Three specific frames and their respective mean magnitudes are highlighted within each trial.

Each video was processed using Matlab R2021a (The MathWorks Inc. 2021). Videos were trimmed at 19,800 frames (11 min) from the last frame to ensure an equal sample size among trials and to allow 4 min of acclimatization at the beginning of each trial. Each frame was then converted to grayscale and optical flow was calculated using the estimatedFlow() function based on the Lucas-Kanade method (Barron et al. 1992). This method calculates the magnitude of the pixels’ flow vector from one video frame to the next. The flow vector consists of the horizontal and vertical components, which indicate displacement of a pixel in the X and Y directions respectively, while the magnitude of the flow vector represents the intensity of the flow at that pixel. For each video frame, magnitude was averaged (mean) across all pixels, yielding one measure of magnitude per frame (N_Frame_ across all trials = 1,801,800) (Fig. 2). While these algorithms do not directly replicate predator motion vision, their ability to detect contrasts in moving prey should correlate with real visual systems, albeit with different detection thresholds that are yet to be well-characterized for guppy predators.

**Fig. 3.**
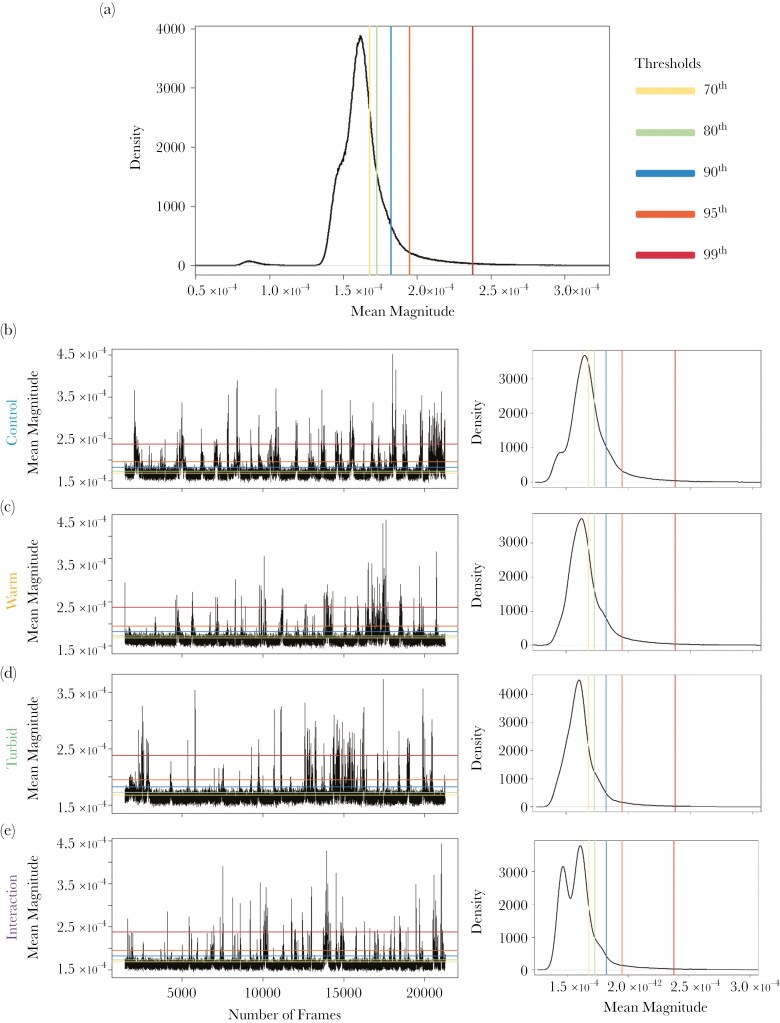
**Thresholds of mean magnitude for the aggregated data**; N = 1,801,800 (**a**). Examples of mean magnitude across one individual trial for the control (**b**), warm (**c**), turbid (**d**) and interaction (**e**) treatment, alongside the mean magnitude distribution for each treatment (N_cont_ = 470,249; N_warm_ = 430,651; N_turb_ = 450,450; N_int_ = 450,450).

Frames where the mean magnitude exceeded a threshold were considered occurrences where the conspicuousness of the fish (ie detectability based on the optical flow) was great enough to be detected by a predator. The statistical analysis was carried out on different datasets which varied in the threshold that was applied, and the results compared qualitatively. The thresholds were calculated as the 99^th^, 95^th^, 90^th^, 80^th^ and 70^th^ percentiles of the mean magnitude pooled across all trials (Fig. 3). We followed this approach to test how general the results were across different thresholds as a sensitivity analysis. Additionally, although the relationship between optical flow as measured in our experiment and detectability to a real predator of the guppy is unknown, varying the threshold could be analogous to comparing predators with varying visual sensitivities. Here, the highest threshold is comparable to predators with lower sensitivity and only able to detect high levels of motion, while the lower thresholds represent predators able to detect subtle prey motion.

For each trial we calculated the proportion of frames where the mean magnitude exceeded the threshold, where a higher proportion indicates trials where fish were detectable more often within the field of view of the camera. The mean magnitude recorded for each frame above the threshold was averaged (mean) per trial, where a greater mean magnitude indicates prey that were more detectable.

Consecutive frames where the mean magnitude exceeded the threshold were grouped as a single “detection event”, which corresponds to the period of time from fish swimming into the visual field of the camera to when they were no longer visible. The number of detection events was then calculated for each trial. Additionally, the mean duration of detection events per trial was calculated to represent the average time available for a predator to respond to the prey.

### Statistical analysis

Each behavioral metric per trial, ie the proportion of frames that exceeded the threshold, mean magnitude within these frames, number of detection events and mean duration of detection events, was included as a response variable in a separate set of generalized linear mixed effect models (GLMMs) using the ‘glmmTMB’ R package v.1.1.7 (Brooks et al. 2017). The models for the proportion of frames were fitted with a beta distribution, the models for the mean magnitude in frames that exceeded the threshold were fitted using a gamma distribution with log link function, the models for the number of detection events were fitted with a negative binomial distribution, and the models for mean duration of detection events a Gaussian distribution. Model assumptions were checked for each distribution type: homogeneity of variance (gamma, Gaussian), dispersion of variance (beta, negative binomial), and normality of residuals (Gaussian) by visual inspection of diagnostic plots (ie QQ and dispersion plots) with R package 'DHARMa' v.0.4.6 (Hartig and Lohse 2022).

A null model was built for each response variable including only the random effect of guppy tank of origin (ie their holding tank). The null model was then compared to seven alternative models each including different explanatory variables: temperature as the only main effect, turbidity as the only main effect, both temperature and turbidity as main effects (Temperature + Turbidity), an interaction term between temperature and turbidity as well as the individual main effects (Temperature × Turbidity), number of guppies included in each trial, trial order (1 to 4) to account for repeated testing (MacGregor and Ioannou 2021), and minutes from midnight to account for diel variations in fish activity levels (Table 1 and 2). To improve interpretability and mitigate the risk of overfitting, we assessed the effects of control variables, ie the number of guppies, trial order, and minutes from midnight, through separate models. By opting for separate models, we aim to achieve clearer insights into the individual effects of control variables on the response variables, maintaining a balance between model simplicity and the need to understand the impact of various factors. The likelihood of each model was then compared using the difference in the Akaike Information Criterion corrected for small sample size (ΔAICc) from the best fitting model. Ranking models with different explanatory variables by their ΔAICc value allows for the inference of which variables drive variation in the response. If the difference in the ΔAICc is greater than 2 units, the model with the smaller ΔAICc value is considered to have strong support as being more likely given the data (Burnham and Anderson 2002). When models fall within 0 to 2 units of the most likely model, it indicates a similar fit. However, if a model with more parameters is within this range of a smaller, more-likely model, it suggests that the similarity in ΔAICc values is primarily due to the addition of an extra parameter, rather than a genuine improvement in fit (Burnham and Anderson 2002). When the null model has the smallest ΔAICc value, it suggests that none of the explanatory variables explain variation in the response. The analysis was repeated on the 5 datasets derived from the different thresholds (the 99^th^, 95^th^, 90^th^, 80^th^ and 70^th^ percentiles of the aggregated mean magnitude values).

**Table 1.** **Model comparisons for the proportion of frames that exceeded the threshold and mean magnitude within these frames.** Each row represents an individual model varying in the explanatory variables: the plus sign indicates multiple variables are main effects, while × indicates an interaction term between temperature and turbidity is included as well as their main effects. Each model also included the random effect of holding tank. ΔAICc: Difference in AIC value corrected for small sample size between each model and the most likely model. df: number of components for each model. In italic are highlighted the null models and in bold the models with greatest support (considering both the ΔAICc and df).

99^th^	ΔAICc	df	95^th^	ΔAICc	df	90^th^	ΔAICc	df	80^th^	ΔAICc	df	70^th^	ΔAICc	df
**Proportion of frames that exceeded the threshold**														
**Repeated Testing**	**0.0**	**4**	**Turbidity**	**0.0**	**4**	Temp. × Turb.	0.0	6	Temp. × Turb.	0.0	6	**Temp. + Turb.**	**0.0**	**5**
Turbidity	7.7	4	Temp. × Turb.	0.7	6	**Temp. + Turb.**	**1.2**	**5**	**Temp. + Turb.**	**0.0**	**5**	Temp. × Turb.	0.3	6
Temp. + Turb.	8.3	5	Repeated Testing	1.3	4	Turbidity	2.3	4	Turbidity	2.5	4	Turbidity	2.7	4
Temp. × Turb.	8.4	6	Temp. + Turb.	1.6	5	Min from Midnight	14.4	4	Min from Midnight	11.9	4	Min from Midnight	12.8	4
Min from Midnight	8.6	4	Min from Midnight	4.9	4	Repeated Testing	19.2	4	Temperature	15.5	4	Temperature	15.1	4
*Null Model*	*13.3*	*3*	*Null Model*	*11.6*	*3*	*Null Model*	*20.4*	*3*	*Null Model*	*16.8*	*3*	*Null Model*	*16.9*	*3*
Temperature	13.8	4	Temperature	13.4	4	Temperature	20.5	4	Repeated Testing	18.4	4	Number of Fish	18.9	4
Number of Fish	14.9	4	Number of Fish	13.7	4	Number of Fish	22.4	4	Number of Fish	18.8	4	Repeated Testing	19.1	4
**Mean magnitude within frames that exceeded the threshold**														
**Temperature**	**0.0**	**4**	**Temperature**	**0.0**	**4**	**Temp. + Turb.**	**0.0**	**5**	**Temperature**	**0.0**	**4**	**Repeated Testing**	**0.0**	**4**
Temp. + Turb.	2.2	5	Temp. + Turb.	1.3	5	Temp. × Turb.	2.1	6	**Repeated Testing**	**0.6**	**4**	Temperature	4.6	4
Temp. × Turb.	4.5	6	Temp. × Turb.	3.5	6	Temperature	3.7	4	Temp. + Turb.	1.3	5	Temp. × Turb.	5.6	6
*Null Model*	*17.1*	*3*	*Null Model*	*26.8*	*3*	Repeated Testing	5.9	4	Temp. × Turb.	2.5	6	Temp. + Turb.	6.1	5
Min from Midnight	18.4	4	Repeated Testing	27.7	4	Turbidity	14.4	4	*Null Model*	*7.5*	*3*	*Null Model*	*9.3*	*3*
Repeated Testing	18.7	4	Turbidity	28.0	4	*Null Model*	*17.7*	*3*	Turbidity	8.6	4	Turbidity	10.7	4
Number of Fish	19.2	4	Number of Fish	28.3	4	Min from Midnight	19.9	4	Min from Midnight	9.6	4	Min from Midnight	11.2	4
Turbidity	19.3	4	Min from Midnight	29.0	4	Number of Fish	19.9	4	Number of Fish	9.6	4	Number of Fish	11.3	4

**Table 2.** **Model comparisons for the number of detection events and mean duration of detection events.** Each row represents an individual model varying in the explanatory variables: the plus sign indicates multiple variables are main effects, while × indicates an interaction term between temperature and turbidity is included as well as their main effects. Each model also included the random effect of holding tank. ΔAICc: Difference in AIC between each model and the most likely model. df: number of components for each model. In italic are highlighted the null models and in bold the models with greatest support (considering both the ΔAICc and df).

99^th^	ΔAICc	df	95^th^	ΔAICc	df	90^th^	ΔAICc	df	80^th^	ΔAICc	df	70^th^	ΔAICc	df
**Number of detection events**														
**Repeated Testing**	**0.0**	**4**	**Turbidity**	**0.0**	**4**	**Turbidity**	**0.0**	**4**	**Turbidity**	**0.0**	**4**	**Turbidity**	**0.0**	**4**
Min from Midnight	4.0	4	Temp. + Turb.	2.0	5	Temp. + Turb.	0.7	5	Temp. + Turb.	0.4	5	Temp. + Turb.	1.7	5
Turbidity	6.6	4	Repeated Testing	3.7	4	Temp. × Turb.	1.9	6	Temp. × Turb.	2.4	6	Min from Midnight	2.8	4
Temp. + Turb.	7.5	5	Min from Midnight	4.0	4	Min from Midnight	21.2	4	Min from Midnight	6.1	4	Temp. × Turb.	4.0	6
Temp. × Turb.	9.5	6	Temp. × Turb.	4.0	6	Temperature	28.2	4	Temperature	9.6	4	*Null Model*	*4.3*	*3*
*Null Model*	*10.7*	*3*	*Null Model*	*12.9*	*3*	*Null Model*	*28.4*	*3*	*Null Model*	*9.6*	*3*	Repeated Testing	5.3	4
Temperature	11.9	4	Temperature	14.6	4	Repeated Testing	30.1	4	Repeated Testing	10.8	4	Temperature	5.8	4
Number of Fish	12.9	4	Number of Fish	14.9	4	Number of Fish	30.6	4	Number of Fish	11.8	4	Number of Fish	6.4	4
**Mean duration of detection events**														
Temp. + Turb.	0.0	5	**Min from Midnight**	**0.0**	**4**	**Repeated Testing**	**0.0**	**4**	**Repeated Testing**	**0.0**	**4**	**Temp. × Turb.**	**0.0**	**6**
**Number of Fish**	**0.2**	**4**	**Turbidity**	**0.7**	**4**	Min from Midnight	23.7	4	Min from Midnight	1.4	4	Temp. + Turb.	4.2	5
**Turbidity**	**0.8**	**4**	Temp. + Turb.	2.9	5	*Null Model*	*26.9*	*3*	Turbidity	2.1	4	Turbidity	4.8	4
Temp. × Turb.	2.3	6	Temp. × Turb.	3.5	6	Temperature	28.9	4	Temp. × Turb.	3.4	6	Min from Midnight	12.3	4
Temperature	2.7	4	Number of Fish	7.2	4	Number of Fish	29.0	4	Temp. + Turb.	4.2	5	Temperature	14.5	4
*Null Model*	*3.2*	*3*	*Null Model*	*7.7*	*3*	Turbidity	29.0	4	*Null Model*	*5.0*	*3*	*Null Model*	*15.2*	*3*
Min from Midnight	3.3	4	Repeated Testing	9.9	4	Temp. + Turb.	31.1	5	Temperature	7.0	4	Repeated Testing	17.4	4
Repeated Testing	4.2	4	Temperature	9.9	4	Temp. × Turb.	33.3	6	Number of Fish	7.2	4	Number of Fish	17.4	4

## Results

**Fig. 4.**
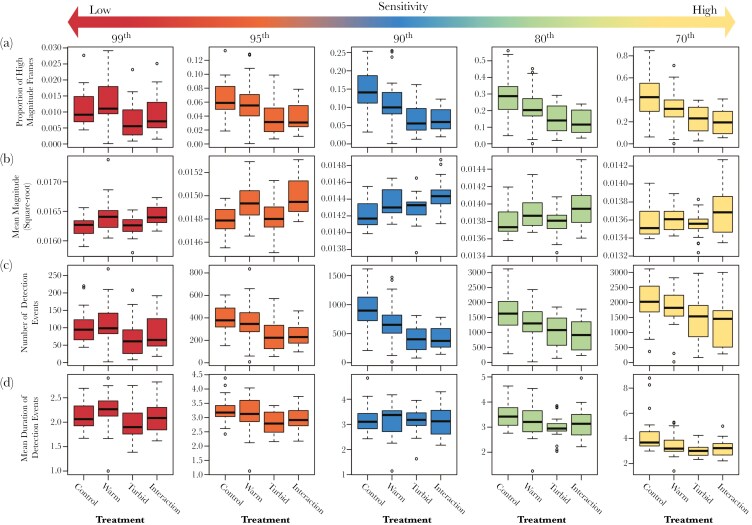
**Response variables as a function of the four experimental treatments** (**a**. Proportion of frames that exceeded the threshold, **b.** Mean magnitude within these frames, **c.** Number of detection events, **d.** Mean duration of detection events): control, warm, turbid and interaction, and derived from the different thresholds; note the different scale of the y axes. In each plot, the horizontal black lines within the boxes represent the median value. The boxes span the interquartile range. The whiskers extend from the most extreme data point by 1.5 times the interquartile range. The black circles represent outliers.

The proportion of frames per trial that exceeded the threshold was influenced mostly by turbidity, but also temperature at the lower thresholds. Models with only turbidity as the main effect (99^th^ and 95^th^ thresholds) and with temperature and turbidity as two main effects (90^th^, 80^th^ and 70^th^ thresholds) were much more likely than the null model (Table 1), although at the 99^th^ percentile the most likely model included repeated testing. For all thresholds, greater turbidity caused a reduction in the proportion of frames exceeding the threshold, suggesting that under these conditions guppies spent a greater time outside the field of view of the camera (Fig. 4a).

Temperature as a sole main effect was the leading predictor for the mean magnitude within frames that exceeded the threshold, as these models had a ΔAICc value of 0 for the 99^th^, 95^th^ and 80^th^ percentile thresholds datasets, and models with temperature were more likely than the null model. In the 90^th^ percentile threshold dataset, the model that included turbidity alongside temperature as additional main effect was the most likely model, and had considerably more support (ΔAICc = 3.7) than the model with temperature as the sole main effect (Table 1). In the 70^th^ percentile dataset, only the model with repeated testing as a main effect was more likely than the temperature-only model. Across thresholds, increases in temperature resulted in greater detectability (higher mean magnitudes) of fish within the view of the camera (Fig. 4b).

The number of detection events was strongly reduced in turbid water. Specifically, the models that included turbidity as the sole main effect had a ΔAICc value of 0 for all thresholds except the 99^th^ percentile (Table 2); here the turbidity-only model was still more likely than the null model. In the interaction treatment, turbidity dominated the response with a similar number of detection events between the turbid and interaction treatments (Fig. 4c).

Compared to the other response variables, there was greater variability between the different threshold datasets for the mean duration of detection events. The greater variability is due to higher thresholds dividing the longer detection events at low thresholds into multiple, shorter ones (Supplementary Figure 1d). The model including test order (ie repeated testing) as the main effect was the most likely for the 90^th^ and 80^th^ percentile thresholds, but it was less likely than the null model for the other thresholds. The model including the time of testing (ie minutes from midnight) was the most likely model for the 95^th^ percentile threshold, and was also supported (ie <2 ΔAICc units from the most likely model with same number of parameters) in the 80^th^ percentile threshold (Table 2). Models that included turbidity had support at all thresholds except the 90^th^ percentile, and in the 70^th^ percentile threshold dataset, there was strong support for the model with the temperature × turbidity interaction term. Increased turbidity reduced the mean duration of detection events (Fig. 4d).

Test order and time of testing (ie repeated testing and minutes from midnight) had a varying degree of influence on the four behavioral responses and for the different threshold datasets (Table 1 and 2). In general, behavioral responses tended to increase with repeated testing (ie greater proportion of frames exceeding the threshold, higher mean magnitude within these frames, and more numerous and longer detection events: Supplementary Figure 2a and b), suggesting some level of habituation to the testing conditions where guppies became more active and approached the video camera more with repeated exposures to the experimental conditions. On the contrary, we observed a decrease in behaviors later in the day, possibly due to a decrease in overall activity with time of day (Supplementary Figure 2c). The number of fish swimming in the experimental arena for each trial did not influence any response, except the mean duration of detection events for the 99^th^ percentile threshold, where more guppies in the arena resulted in longer durations (Supplementary Figure 2c).

Lowering the threshold from the 99^th^ to the 70^th^ percentile increased the number of detection events almost 18-fold, from 8,461 to 149,767. This is consistent with the increase in the number of frames exceeding the threshold, which rose from 18,215 to 540,578. However, the most important predictors for the four response variables were relatively constant across the thresholds used (Table 1 and 2), supporting the consistency of the findings

## Discussion

Our findings are consistent with previous research (Turesson and Brönmark 2007; Jönsson et al. 2013; Chaparro-Herrera et al. 2020) suggesting that due to the visual impairment caused by turbid water, the proportion of time that prey were in close enough proximity to be detected by a predator decreased. Our study also suggests that once prey are close enough, recognizing, targeting and capturing prey may become more difficult due to detection events being shorter in duration. However, we show that when detection events occurred in warmer water, prey detectability (ie the magnitude of the optical flow) was greater compared to the control and turbid treatments, likely due to the increased activity of the prey when within the camera’s field of view (Krause and Godin 1995). These results suggest that in aquatic habitats that are becoming warmer and more turbid, warmer temperatures may, at least partially, compensate for the negative effects of turbidity on visual predators.

Predation risk can increase with warmer temperatures as ectothermic predators experience enhanced kinematics and greater predation drive (ie hunger levels caused by increased metabolic rates, Domenici et al. 2019). In response to increased threat, prey often reduce their activity levels to reduce their detectability (Eilam 2005; Ajemian et al. 2015). However, prey fish are also subject to higher metabolic rates in warmer water (Bartolini et al. 2015). As a result, increased activity levels have been observed in prey fish under warmer conditions, even in the presence of a predator. Guppies show increased shoaling behavior as a mitigating anti-predator strategy (Weetman et al. 1999). Although more aggregated prey can reduce encounter rates (Ioannou et al. 2011; Johannesen et al. 2014), our experiment did not find evidence supporting a change in the number or duration of detection events driven by temperature. A parallel study by Allibhai et al. (2023), which assessed the social behavior of the guppies in this experiment using video recordings from above the experimental tank (measuring aggregation metrics and refuge use), reported an increase in shoaling tendencies at higher temperatures, but only at a local scale where the guppies were closer to their nearest neighbors in warmer water. There was no evidence from this study, however, that the distance between the shoals was affected by temperature, and the distance between shoals has been shown to be a more important factor in determining encounter rate than shoal size (Ioannou et al. 2011).

Our findings suggest that the frequency that prey were visible to the camera was dominated by the effects of turbidity, rather than by temperature. This is a comparative effect, ie where the response was dominated by only one of the multiple stressors (Folt et al. 1999). Previous research on guppy activity levels under turbid conditions has yielded mixed results, with some studies indicating decreased activity due to a heightened risk perception (Borner et al. 2015), while other studies show that turbidity has no influence on guppy activity levels (Zanghi et al. 2023), although this may be due to different levels of turbidity used in these experiments (eg 15 versus 700 NTU). Furthermore, observations by Allibhai et al. (2023) indicated that the increase in turbidity likely did not affect the risk perception of the guppies in this experiment, as refuge use remained unchanged. Nevertheless, the guppies exhibited reduced aggregation in turbid conditions, possibly due to the visual constraints of detecting distant shoal mates (MacGregor and Ioannou 2023). While this reduction in aggregation could increase encounter rates (Ioannou et al. 2011; Johannesen et al. 2014), our findings suggest that this effect was outweighed by the direct effect of turbidity reducing the viewing distance from our hypothetical predator. In this context, a prey population that is more evenly distributed may have individuals closer to a predator compared to aggregated prey, yet remain undetected.

Quantifying the detectability of moving prey is more challenging compared to studying static prey, which are typically used in research on animal coloration (Stevens et al. 2007; Troscianko and Osorio 2023). This complexity arises from the dynamic nature of motion, involving varying speed and changes in direction, which can influence detectability by predators (Cuthill et al. 2019). In our study, the overall number of detection events was greatly reduced (~18 fold) with lower visual sensitivity (ie higher threshold of magnitude). This highlights the importance of considering different species’ visual abilities in a multi-predator system. However, modeling non-human visual abilities (such as spatial acuity, contrast sensitivity, and movement sensitivity) is also challenging (Caves and Johnsen 2018). Our method of using optical flow to measure detectability offers several advantages, as optical flow algorithms capture the motion dynamics of animals, providing a continuous and objective measure of movement intensity. This approach can be valuable for predicting predator-prey interactions in more realistic settings. While there is still much to learn about the specific visual sensitivities of different predators (Heathcote et al. 2020), our method can incorporate species-specific visual acuity data to be tailored to specific systems.

An even greater challenge is to consider the impacts of environmental stressors on detection by predators that rely on sensory modalities other than vision. Indeed, the guppy is prey to the wolf fish *Hoplias malabaricus* and the freshwater shrimp *Macrobrachium crenulatum*, which are crepuscular/nocturnal, and use olfactory and tactile cues to sense their prey (Magurran 2005; Croft et al. 2006). In the study of Ehlman et al. (2019), the density of the pike cichlid *Saxatilia frenata* was lower in more turbid water as was their success in capturing guppies, while *H. malabaricus* was unaffected. Across species, visual predators tend to be affected more negatively by turbidity than non-visual predators (Zanghi and Ioannou 2024). However, while cameras are effective at quantifying how prey may appear to predators, especially with appropriate calibration and visual modeling, equivalent technology to measure the olfactory or tactile cues of prey is far less accessible, especially dynamically when prey are in motion as in our study. This makes the study of non-visual prey cues particularly challenging, including how these cues are affected by environmental stressors. Other guppy predators, such as the blue acara *Andinoacara pulcher*, use pursuit as well as ambush to capture their prey, and again, studying how environmental stressors impact these predators is constrained by technological limitations (Szopa-Comley and Ioannou 2022).

As water temperature and turbidity increase in aquatic habitats globally due to human activities (eg quarrying (Ehlman et al. 2019), tilling (Dieter 1991), agricultural eutrophication (Egertson et al. 2004), extreme weather events (Mori et al. 2019; Chiang et al. 2021), and global warming (Valipour et al. 2021)), there is a strong need to integrate behavioral studies with scenarios that better represent realistic natural conditions. The inclusion of multiple stressors in behavioral ecology has been increasing in current research. It is estimated that up to one-quarter of studies find interactions between stressors (ie synergies and antagonisms), in contrast to additive effects (Darling and Côté 2008). Antagonistic interactions are more common than synergies, particularly in freshwater habitats (Darling et al. 2010; Jackson et al. 2016). While antagonistic interactions can be beneficial to impacted ecosystems due to mitigating effects, the overall impact can still be negative compared to pristine conditions (Vinebrooke et al. 2004; Orr et al. 2020). Our study did not observe interactions between stressors impacting the same response variable (eg duration of detection events). However, we found mitigating effects of increased temperature and turbidity on different metrics (ie mean magnitude and number of detection events) that together determine the detectability of prey.

Understanding shifts in animal behavior as a result of environmental change can be a valuable monitoring tool (Bro-Jørgensen et al. 2019). Behavioural changes at an individual level can serve as early indicators of ecological stress, often leading to altered population dynamics and reproductive success (Candolin 2009). Therefore, comprehensive and ecologically realistic approaches are important to inform the effective management and conservation of local natural resources.

## Supplementary Material

araf079_suppl_Supplementary_Figures

## Data Availability

Analyses reported in this article can be reproduced using the data provided by Zanghi et al. (2025).
