# Supplementary material for: Enhanced conspicuousness of prey in warmer water mitigates the constraint of turbidity for predators
Source: Behav Ecol. 2025 Jul 10;36(4):araf079. doi: 10.1093/beheco/araf079 (PMC12288029; doi:10.1093/beheco/araf079)
Supplement: araf079_suppl_Supplementary_Figures [file araf079_suppl_supplementary_figures.docx]

**Supplementary Figures**


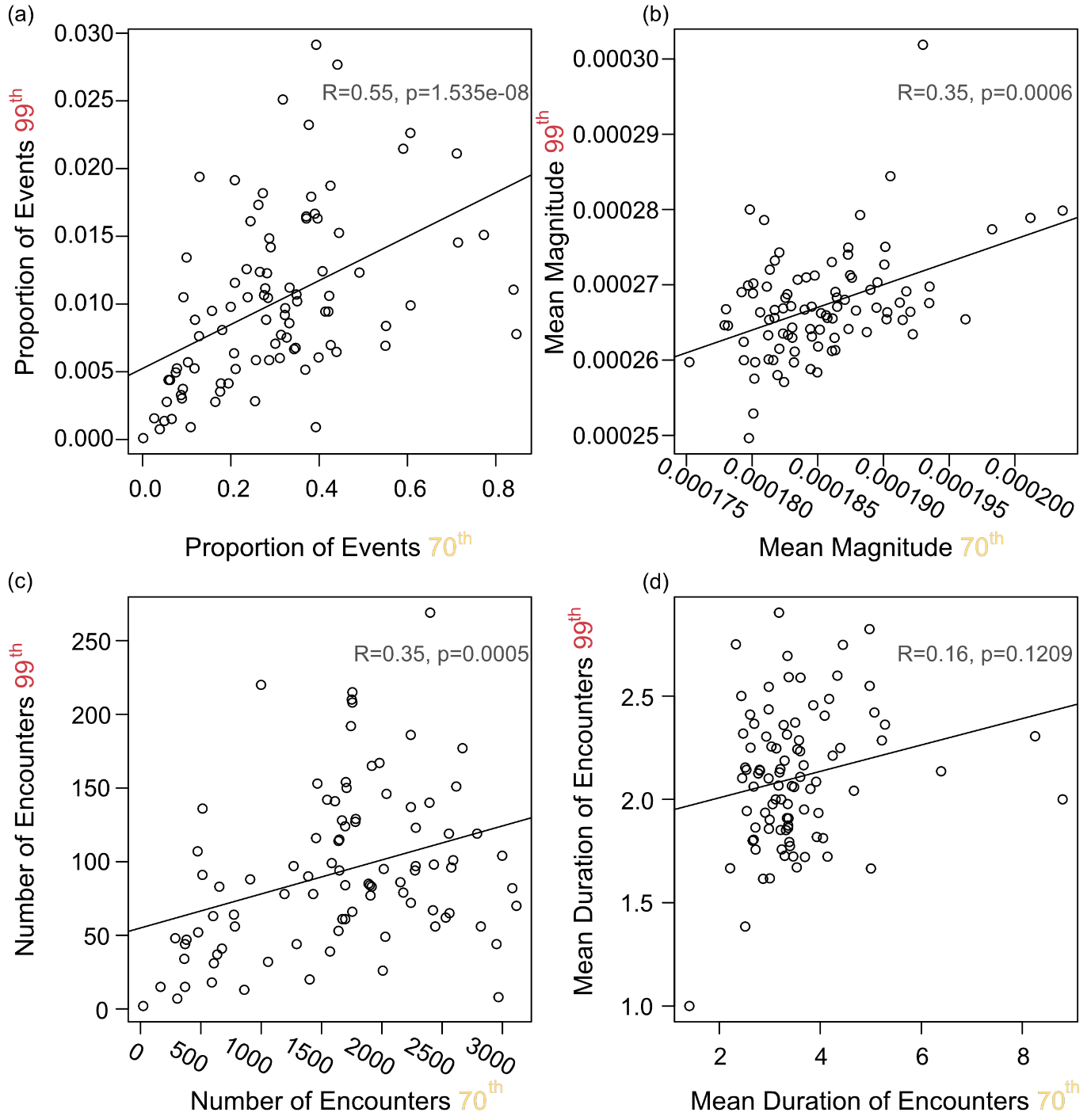


**Supplementary Figure 1.** Relationship between the highest (99th percentile) and lowest (70th percentile) sensitivity thresholds for each response variable: (a) proportion of frames that exceeded the threshold, (b) mean magnitude within these frames, (c) number of detection events, (d) mean duration of detection events (N=91). Regression lines, Spearman’s rank correlation coefficient (R) and significance of relationship (p) are reported for each response.


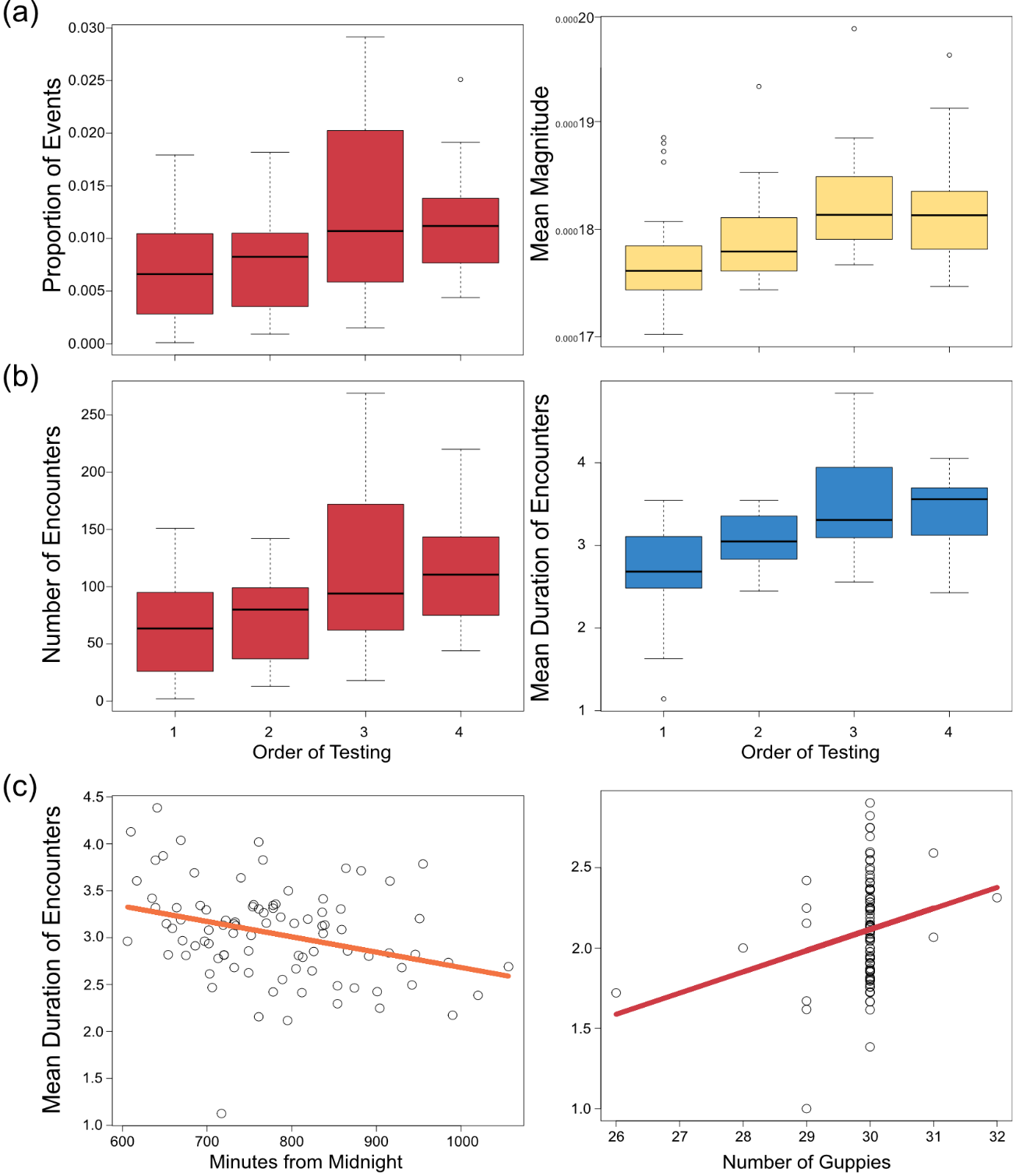


**Supplementary Figure 2.** Response variables (a. left: proportion of frames that exceeded the threshold, a. right: mean magnitude within these frames, b. left: number of detection events, b. right: mean duration of detection events) as a function of the order of repeated testing (1 to 4), for the 99th (a. & b. left), 70th (a. right) and 80th (b. right) percentiles dataset. In each plot the horizontal black lines within the boxes represent the median value. The boxes span the interquartile range. The whiskers extend from the most extreme data point by 1.5 times the interquartile range. The black circles represent outliers. The mean duration of encounters as a function of (c) minutes from midnight (left, 95th percentile threshold) and number of guppies used in the trial (right, 99th percentile threshold). For each plot, dots represent individual observations (N=91), the lines represent the predicted values calculated from the GLMM coefficients. Note the different scale of the y axes across the different thresholds.
